# Effect of Propolis Extracts on OxLDL and LOX-1 Levels in ApoE Knockout Mice Fed a High Fat Diet

**DOI:** 10.3390/life15040565

**Published:** 2025-03-31

**Authors:** Katip Korkmaz, Orhan Deger, Ertugrul Yigit, Hüseyin Avni Uydu, Tolga Mercantepe, Selim Demir

**Affiliations:** 1 Department of Nutrition and Dietetics, Faculty of Health Science, Karadeniz Technical University, 61080 Trabzon, Turkey; selim.demir@ktu.edu.tr; 2 Department of Medical Biochemistry, Faculty of Medicine, Karadeniz Technical University, 61080 Trabzon, Turkey; odeger@ktu.edu.tr (O.D.); ertugrulyigit@ktu.edu.tr (E.Y.); 3 Department of Medical Biochemistry, Faculty of Medicine, Samsun University, 55080 Samsun, Turkey; huseyin.uydu@samsun.edu.tr; 4 Department of Histology and Embryology, Faculty of Medicine, Recep Tayyip Erdogan University, 53020 Rize, Turkey; tolga.mercantepe@erdogan.edu.tr

**Keywords:** ApoE^−/−^ mice, atherosclerosis, LOX-1, oxidized LDL, propolis

## Abstract

Atherosclerosis, which has important effects on the development of cardiovascular diseases, is a widespread health problem with the highest mortality rate globally. In this study, we aimed to assess the impact of water and ethanolic extracts of propolis on oxidized low-density lipoprotein (OxLDL) and lectin-like oxidized low-density lipoprotein receptor-1 (LOX-1) in the progression of the atherosclerotic process, which is characterized by oxidative stress, inflammation, and dyslipidemia. In our study, apolipoprotein E knockout (ApoE^−/−^) and C57BL/6J mice were used as study groups. Water (WEP) and ethanolic extracts (EEP) of propolis were administered intraperitoneally to ApoE^−/−^ and C57BL/6J mice modeled with a high-fat diet. Under anesthesia, the animals were euthanized by decapitation, and serum, along with aortic tissues, was collected. Serum total cholesterol (TC), triglyceride (TG), OxLDL and LOX-1 levels, OxLDL levels in aortic tissue homogenate, and subendothelial lipid accumulation levels by histological staining were determined in mice and statistical analyses were performed. WEP and EEP supplementation significantly decreased serum TC, TG, OxLDL, LOX-1, and tissue OxLDL levels and reduced plaque burden in the aortic root, with statistically significant differences observed. Those results suggest that propolis extracts have a potential treatment option for atherosclerosis, as a food supplement or a complementary medical/functional food. However, further research is needed to elucidate their molecular mechanisms, evaluate clinical efficacy and safety, and explore possible synergistic effects with existing atherosclerosis treatments.

## 1. Introduction

Atherosclerosis is a disease characterized by chronic inflammation and accumulation of lipid-rich plaques in arterial walls and has important implications in cardiovascular diseases (CVD) [1]. The development of the atherosclerotic process begins with endothelial dysfunction and leads to structural changes in the arterial wall due to many cardiovascular risk factors such as dyslipidemia, hypertension, and diabetes. These functional disorders promote inflammation and oxidation and lead to the migration of inflammatory cells into the subendothelial area [2,3]. The production of reactive oxygen species (ROS) and lipid oxidation in the vasculature play a crucial role in the development of atherosclerotic CVD. ROS cause endothelial dysfunction by reducing the anti-inflammatory and anti-atherogenic effects of nitric oxide and activating pro-inflammatory gene expression [3]. Moreover, ROS serves as a powerful activator of matrix metalloproteinases within signaling cascades, triggering vascular pro-inflammatory and pro-thrombotic gene expression through the activation of the transcription factor nuclear factor kappa B (NF-κB). In the atherosclerotic process, oxidized low-density lipoproteins (OxLDL) formed by the oxidation of low-density lipoproteins (LDL) in the arterial wall cause early visible atherosclerotic lesions known as fatty streaks, leading to foam cell formation [2,4]. Foam cell formation is increased by interactions between macrophages and smooth muscle cells (SMCs). Macrophages directly increase lipid or cholesterol accumulation within SMCs, and SMCs also promote macrophage-foam cell formation through extracellular matrix components. This bidirectional interaction leads to cellular complexity in atherosclerotic lesions [5]. Apolipoprotein E (ApoE) plays a crucial role in lipid metabolism by regulating cholesterol homeostasis and preventing atherosclerosis; however, in the absence of ApoE (ApoE^−/−^), LDL oxidation increases, lectin-like oxidized low-density lipoprotein receptor-1 (LOX-1) expression is upregulated, and inflammation is triggered, leading to an accelerated atherosclerotic process [6,7]. OxLDL causes important effects such as endothelial dysfunction, inflammation, and foam cell formation in the atherosclerotic process [8,9,10]. LOX-1, which acts as an OxLDL receptor, is effective in the atherosclerotic process. LOX-1 is primarily expressed in endothelial cells and mediates OxLDL uptake, leading to endothelial dysfunction and the formation of foam cells, which are the main components of atherosclerotic plaques. LOX-1 also plays a role in ROS formation and accelerates the atherosclerotic process [11,12]. These processes, driven by oxidative stress and lipid metabolism, lead to the development of atherosclerotic lesions. As a result, atherosclerotic plaques are formed by the accumulation of immune cells, SMC proliferation, and extracellular matrix deposition. As these processes progress, plaques can rupture and lead to thrombosis, blocking blood flow and causing serious cardiovascular consequences such as myocardial infarction [13,14]. Prevention of CVD requires early identification of risk factors and therapeutic interventions. In CVD, it is necessary to control modifiable risk factors such as lifestyle changes, smoking, hypertension, dyslipidemia, obesity, and a sedentary lifestyle, and if these factors are present in individuals, efforts should be made to slow down disease progression [15,16]. Many properties of natural products, especially their antioxidant and anti-inflammatory effects, show important potential in preventing CVD [17]. Additionally, with the increasing side effects of synthetic chemical drugs, the use of traditional natural products has risen [18]. Propolis, a traditional natural bee product, has attracted interest as a promising agent in the prevention and treatment of CVD. Propolis is a resinous natural bee product rich in phenolic compounds collected from the buds and bark of trees by honey bees (*Apis mellifera*) [19,20]. Propolis is valued for its anti-inflammatory, antioxidant, and lipid-lowering properties, attributed to its rich composition of bioactive compounds, including flavonoids, phenolic acids, and terpenoids [21,22,23]. Although numerous studies have explored the general antioxidant and anti-inflammatory properties of propolis, few have specifically investigated its direct effects on key atherosclerotic biomarkers such as OxLDL and LOX-1, particularly within a genetically susceptible model like ApoE^−/−^ mice [24,25]. Despite its rich bioactive content, the bioavailability of propolis is relatively low due to metabolic transformations of flavonoids and phenolic compounds in the gastrointestinal tract. Therefore, extraction methods such as ethanolic extraction are commonly employed to enhance the absorption and biological efficacy of its active constituents [24]. However, studies on the effects of propolis extracts on oxidative stress, inflammation, and dyslipidemia in the development of atherosclerosis remain limited in the literature. We hypothesized that water and ethanolic extracts of propolis would attenuate atherosclerotic progression in ApoE^−/−^ mice fed a high-fat diet (HFD) by reducing oxidative stress and inflammation through modulation of OxLDL and LOX-1 levels. In this study, we aimed to demonstrate the effects of propolis against the development of the atherosclerotic process and the potential effects of natural bee products as a complementary treatment to current therapies. For this purpose, the potential effects of propolis extracts on inflammatory and oxidative stress biomarkers (OxLDL and LOX-1) in atherosclerosis, as well as atherosclerotic plaque development in ApoE^−/−^ mice fed a HFD, were investigated using both biochemical and histological methods.

## 2. Materials and Methods

### 2.1. Chemicals, Diets and ELISA Kits

Triton X-100 (9036-19-5), Oil Red O (ORO; 102419), and phosphate-buffered saline (PBS; 524650-1EA) used in the experimental procedures were supplied by Sigma-Aldrich (St. Louis, MO, USA). Atherogenic (high-fat, high-cholesterol) diet (D12108C, New Brunswick, NJ, USA) and the control diet (D12104C, New Brunswick, NJ, USA), specifically formulated for rodent models, were obtained from Research Diets. Quantification of OxLDL and LOX-1 levels was performed using commercially available enzyme-linked immunosorbent assay (ELISA) kits provided by FineTest, Palm Coast, FL, USA and Thermo Fisher Scientific, Waltham, MA, USA, respectively, following the protocols recommended by the manufacturers.

### 2.2. Experimental Animals

All procedures involving animals were conducted in accordance with ethical standards and approved by the Ethics Committee of the Faculty of Medicine at Karadeniz Technical University (KTU; Approval No: 2022-23). The experimental design followed the guidelines established by the National Institutes of Health for the care and use of laboratory animals and was prepared in accordance with the ARRIVE guidelines to ensure transparency and reproducibility in animal research [26,27]. ApoE^−/−^ mice, due to their genetic predisposition, are widely used in studies related to CVD, especially atherosclerosis and obesity, as they spontaneously develop atherosclerotic plaques [28]. C57BL/6J wild-type mice (RRID: IMSR JAX:000664) and ApoE^−/−^ mice (RRID: IMSR JAX:002052), bred at the Surgical Research Center of KTU, were used in the study. Mice were purchased at 6–8 weeks of age and randomly allocated into experimental groups. Throughout the study, the animals were maintained under controlled environmental conditions (22–23 °C, 50–60% relative humidity) with a 12-h light/dark cycle. They had ad libitum access to food and water, and their health status was monitored regularly to ensure animal welfare and data reliability.

### 2.3. Preparation of Water and Ethanolic Extracts of Propolis

Propolis samples used in this study were collected from different parts of Turkey, representing the northern (Trabzon), eastern (Erzurum), western (Zonguldak), and southern (Adıyaman) regions, and were homogenized based on their chemical composition to produce a single, standardized extract. This strategy was employed to increase the diversity of bioactive compounds and improve the generalizability of the findings [29]. The homogenized samples were weighed in specific ratios, and distilled water was used to prepare the water extract of propolis (WEP), while 30% ethanol was used for the ethanolic extract of propolis (EEP). The mixtures were vortexed and incubated in a shaker at 60 °C and 150 rpm for 24 h to ensure dissolution. Subsequently, the extracts were filtered through filter paper and sequentially passed through 0.45 µm and 0.22 µm membrane filters. The final extracts were stored at +4 °C in the dark until use.

### 2.4. Study Groups, Treatment Administration, and Sample Collection Procedures

The total number of animals used in the experiment was determined using G*Power 3.1 software, based on an effect size of 0.60, alpha error of 0.05, power of 0.85, and a total of six experimental groups, resulting in a minimum sample size of 48. Each group consisted of eight randomly assigned male mice (Table 1).

The experimental design included both control C57BL/6J and ApoE^−/−^ mice, the latter used to model atherosclerosis. Mice in the WEP and EEP groups were fed a HFD for 16 weeks, followed by daily i.p. administration of 400 mg/kg WEP or 200 mg/kg EEP for the last four weeks. Ethanol, used as the solvent for EEP, was also administered i.p. to a separate group for comparison. All propolis solutions were prepared in advance and stored under appropriate conditions until use. EEP was extracted using 30% ethanol, while WEP was prepared using distilled water. Throughout the dietary intervention, animals were fed 3–6 g of feed per day and weighed at four-week intervals to monitor weight changes. At the end of the 16-week study period, animals were anesthetized with ketamine (80 mg/kg) and xylazine (10 mg/kg), then euthanized by decapitation. Blood samples were collected into gel-separation tubes, and sera were stored at −80 °C until biochemical analysis. For tissue analysis, the aortic root and aortic arch areas most prone to atherosclerotic plaque formation were carefully dissected. Aortic root tissues were fixed in preparation for histological examination, while aortic arch samples were frozen at −80 °C for further biochemical assessments [30].

### 2.5. Serum Lipid Profile Analysis

Serum triglyceride (TG) and total cholesterol (TC) levels in mice were measured using enzymatic colorimetric methods on the Beckman Coulter AU5800 (Brea, CA, USA) clinical chemistry analyzer at the Medical Biochemistry Laboratory of KTU.

### 2.6. Quantification of Serum OxLDL and LOX-1 Levels

The concentrations of serum OxLDL (EM0400, FineTest, Wuhan, China) and LOX-1 (EM50RB, Thermo Fisher) were quantified using specific commercial ELISA kits, as per the manufacturers’ instructions. For each assay, 100 µL of serum sample was dispensed into designated wells, along with corresponding standard solutions. A sandwich ELISA protocol was followed, and absorbance readings were obtained at 450 nm using a Spectra-Max Paradigm plate reader (Molecular Devices, San Jose, CA, USA). The outcomes were reported in nanograms per milliliter.

### 2.7. Preparation and Processing of Aortic Arch Tissue for Protein Analysis

The aortic arch tissue was immersed in PBS containing 0.01% Triton X-100 and homogenized on ice for 2 min at 6000 rpm. This was followed by sonication for 20 s at 130 watts and 20 kHz using a Sonics-Vibracell sonicator (Newtown, CT, USA). The homogenates were subsequently centrifuged at 15,000× *g* for 15 min using a Beckman Coulter Allegra™ 64R centrifuge (Brea, CA, USA), and the resulting supernatants were carefully collected. Protein content was then determined using a commercial bicinchoninic acid assay kit (UNSPSC Code: 12352106).

### 2.8. Quantification of OxLDL Levels in Aortic Arch Tissue

OxLDL levels in the aortic arch tissue were quantified using a specific commercial sandwich ELISA kit (EM0400, FineTest, Wuhan, China). For the assay, 100 µL of tissue supernatant and standard solutions were added to antibody-coated ELISA plates. The plates were incubated and washed according to the manufacturer’s instructions. Colorimetric detection was carried out at 450 nm using a Spectra-Max Paradigm (San Jose, CA, USA) microplate reader. OxLDL concentrations were calculated based on the absorbance values and expressed as ng per mg of protein.

### 2.9. Histological Examination of Aortic Root Tissue

For histological evaluation, the aortic root tissues were initially trimmed to a size of approximately 1.5 cm^3^ and stored at −80 °C for 10 min. Tissue sections with a thickness of 6–8 µm were prepared using a cryostat (Leica 3050S, Leica Biosystems, Nußloch, Germany) at −18 °C, embedding the samples in a suitable freezing medium (Sigma-Aldrich, Darmstadt, Germany). The sections were subsequently stained with ORO following the manufacturer’s protocol to visualize lipid-rich areas. Counterstaining was performed using Mayer’s hematoxylin. Prepared slides were examined and imaged using a digital microscope system (Olympus BX51, Olympus Corp, Tokyo, Japan) equipped with a digital camera (Olympus DP71, Olympus Corp, Japan). Histological assessment was semi-quantitatively performed by evaluating adipocyte subendothelial involvement, adipocyte vascular wall involvement, fibrous cap formation, and the overall aortic histopathological score [31].

### 2.10. Statistical Evaluation

All statistical analyses were performed using SPSS 23.0 statistics version. The Kolmogorov–Smirnov test was initially used to assess the normality of data distribution. Body weight changes were normally distributed (*p* < 0.05) and are presented as mean ± standard deviation (SD). Comparisons among multiple groups were carried out using one-way ANOVA followed by Tukey’s post-hoc test. For data not conforming to normal distribution, results were presented as median and interquartile range (IQR), and comparisons between groups were conducted using the Mann–Whitney U test. A Bonferroni correction was applied for multiple comparisons, with statistical significance set at α = 0.05/12 = 0.004. Histological data, evaluated semi-quantitatively, were analyzed using non-parametric tests. The Shapiro–Wilk test, Q-Q plots, skewness–kurtosis values, and Levene’s test were applied to assess data normality and homogeneity. The Kruskal–Wallis test, followed by Tamhane’s T2 post-hoc test, was used where appropriate. Statistical significance for histological analyses was considered at *p* < 0.05. Concentrations for biochemical parameters were calculated via Four-Parameter Logistic Regression using standard curves generated through www.myassays.com, and graphical representations were created using Microsoft Excel 2023.

## 3. Results

### 3.1. Extracts of Propolis Reduce Weight Gain of Mice

**Figure 1:**
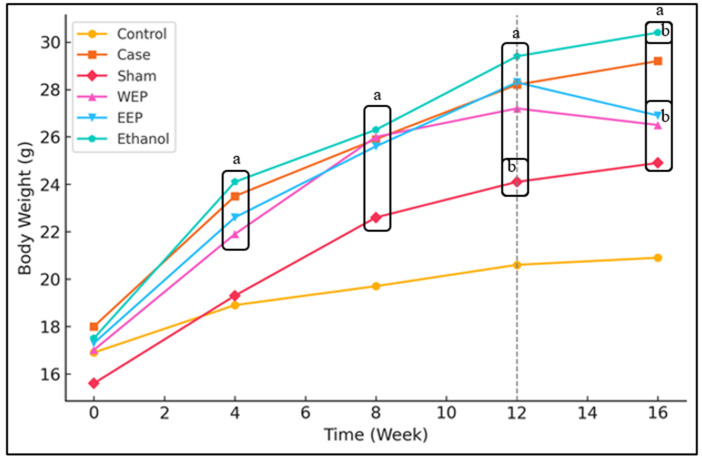
Results are presented as mean ± SD. a: Statistically significant difference compared to the Control group (*p* < 0.05), b: Statistically significant difference compared to the Case group (*p* < 0.05).

Propolis extracts were administered to mice with HFD to determine weight changes every four weeks during the experiment. The measurement results were shown in Figure 1. There was no statistically significant difference in the weights of the groups at the beginning of the experiment (*p* > 0.05). However, weight changes were observed between the groups in the following weeks. After the 4th, 8th, and 12th weeks, the weight values of the Case, WEP, EEP, and Ethanol groups were significantly higher than the Control group (*p* < 0.05). There was no significant difference between these groups (*p* > 0.05). The weights of the Sham group were significantly higher than the Control group at the end of the 8th, 12th, and 16th weeks (*p* < 0.05). The weights of the WEP and EEP groups were significantly lower (*p* < 0.05) than the Case and Ethanol groups at the 16th week after 4 weeks of injection, whereas a significantly higher difference was observed in the Control and Sham groups (*p* < 0.05).

### 3.2. Extracts of Propolis Reduce HFD-Induced Dyslipidaemia

The routine biochemistry results measured in the sera of the mice used in the experiment are given in Table 2. HFD-induced TC and TG levels were significantly higher in ApoE^−/−^ mice than in mice fed CD. At the end of the 16-week experimental period, both TC and TG levels of WEP and EEP groups were significantly lower (*p* < 0.004) than the Case group. TG levels of WEP and EEP groups were significantly lower (*p* < 0.004) than the Control group, while TC levels were significantly higher (*p* < 0.004) than the Control group.

### 3.3. Extracts of Propolis Reduce Oxidative Stress Markers

**Figure 2:**
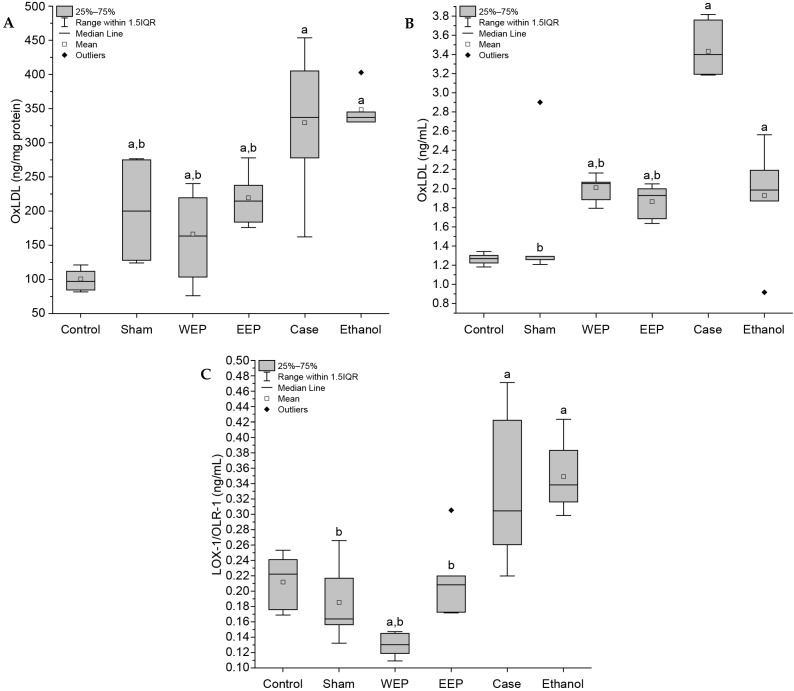
(**A**) Aortic arch OxLDL levels, (**B**) Serum OxLDL levels, (**C**) Serum LOX-1 levels. a: Statistically significant difference compared to the Control group (*p* < 0.004), b: Statistically significant difference compared to the Case group (*p* < 0.004).

Serum OxLDL and LOX-1 levels, which have important roles in the atherosclerotic process, and tissue aortic arch OxLDL levels are shown in Figure 2. A statistically significant difference (*p* < 0.004) was observed in OxLDL and LOX-1 levels in WEP and EEP groups compared to the Case group.

### 3.4. Attenuation of Aortic Root Plaque Formation and Lipid Deposition by Propolis Extracts

**Figure 3:**
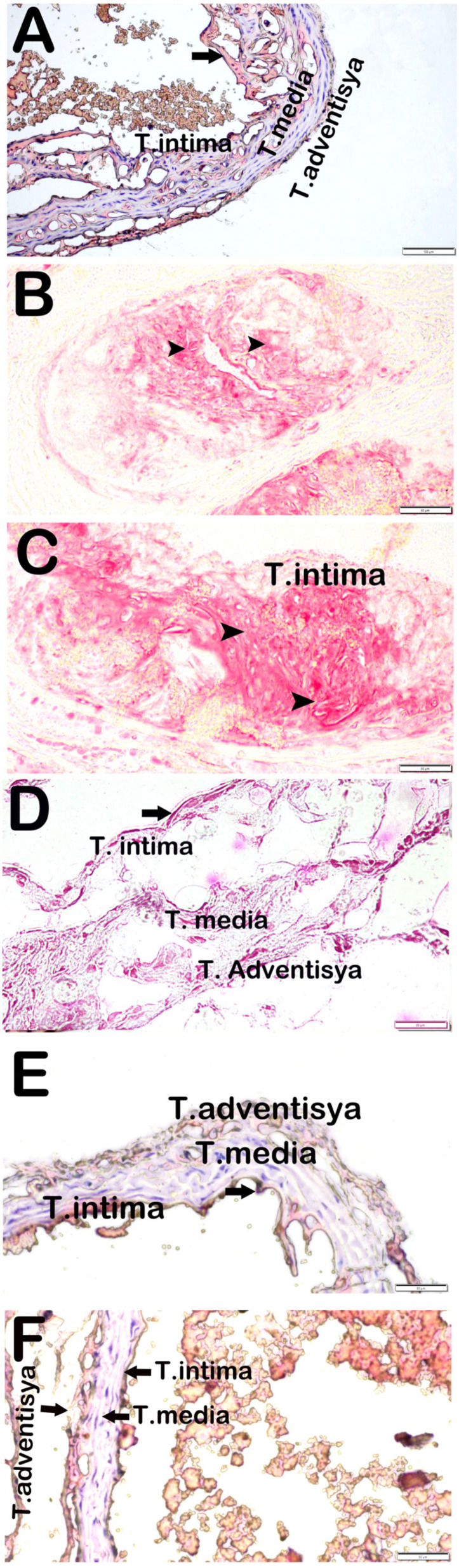
Representative light microscopy pictures of aortic root tissue obtained by cryostat method stained with ORO. (**A**) (×20) Control: In the aortic root tissue sections, the endothelium and subendothelial connective tissue within the tunica intima layer exhibit a normal structure (arrow). Additionally, the tunica media and adventitia layers also maintain a normal structural appearance. (**B**) (×20) Sham: In the aortic root tissue sections, adipocyte accumulations are observed in the subendothelial area of the tunica intima layer (arrowhead). Additionally, adipocyte accumulations are present in certain regions of the tunica media (arrowhead). (**C**) (×20) Case: Aortic root tissue sections show dense adipocyte accumulations in the tunica intima and media (arrowhead). (**D**) (×20) WEP: Aortic root tissue sections show decreased adipocyte accumulation in the subendothelial area (arrow). (**E**) (×20) EEP: In aortic root tissue sections, it is seen that adipocytes are decreased in the subendothelial area (arrow). (**F**) (×20) Ethanol: Aortic root tissue sections show dense adipocyte accumulation in the tunica intima and media layer (arrow).

In ApoE^−/−^ mice subjected to a HFD, both atherosclerotic plaque burden and subendothelial lipid accumulation were markedly elevated when compared to the Control group (*p* < 0.05). Notably, administration of propolis extracts during the final four weeks of the feeding period significantly mitigated plaque development and lipid deposition in the aortic root (*p* < 0.05), as illustrated in Figure 3.

Histopathological damage scores evaluated under the categories of adipocyte subendothelial involvement, vascular wall involvement, and fibrosis at the aortic root level are summarized in Table 3. While the median scores for all parameters were zero in the control group, significant increases were observed in the Case group (*p* < 0.05). Notably, adipocyte subendothelial involvement was the highest in the Case group.

## 4. Discussion

Atherosclerosis is characterized by chronic inflammation and oxidative stress, with OxLDL playing a central role in its progression. OxLDL and its receptor LOX-1 accelerate atherosclerotic plaque formation in inflammatory processes [32]. To reduce the role of OxLDL, which has important effects in the atherosclerotic process, prevent lipid accumulation in the vascular wall, and slow down disease progression, treatments aimed at removing OxLDL from vascular circulation are needed [33]. Studies show that propolis, one of the natural bee products, may have anti-inflammatory and anti-atherogenic effects by regulating OxLDL levels and may potentially have therapeutic effects against atherosclerosis [34,35]. In this study, we aimed to investigate the effect of water and ethanolic extracts of propolis on OxLDL and LOX-1 levels in HFD-fed ApoE^−/−^ mice. A model of atherosclerosis was established in ApoE^−/−^ mice fed HFD. TC, TG, OxLDL, and LOX-1 levels, as well as atherosclerotic plaque burden and subendothelial lipid accumulation, were significantly increased in the Case group (*p* < 0.004). However, i.p. injection was started in the WEP and EEP groups at week 12, and these groups showed significant weight loss at the end of week 16. A statistically significant difference was observed when WEP and EEP groups were compared with the Case group (*p* < 0.004). When the weights of the WEP and EEP groups were compared before and after injection, they experienced a weight loss of 3.03% and 6.64%, respectively. In the literature, it has been reported that propolis extracts reduce body weight, visceral adipose tissue weight, serum TG and TC levels in mice, and reduce HFD-induced dyslipidemia by downregulating the expression of genes related to lipid metabolism. Additionally, propolis has been shown to decrease the accumulation of saturated fatty acids in the liver and skeletal muscle while increasing their excretion through feces, ultimately leading to reduced fat absorption and increased fecal weight. However, no study was found in which WEP had a direct effect on animal weight. In this respect, our results align with existing data in the literature [36,37,38,39,40]. Since fatty streaks formed by foam cells in mice fed an atherogenic diet were observed from the 10th to 12th week of the diet, i.p. injection was started at these times in the WEP and EEP groups [41]. Additionally, aortic tissues were obtained from mice at regular intervals, and fatty streak development was examined under a microscope. To observe plaque stabilization or reduction in the atherosclerotic lesion area in ApoE^−/−^ mice, water and ethanolic extracts of propolis were administered to WEP and EEP groups by i.p. injection every day for the last 4 weeks after the 12th week, in addition to the diet. The dosage was determined as 400 mg/kg and 200 mg/kg for the WEP and EEP groups, respectively, considering both the number of animals and the amount of consumables required [31,41]. The choice of 30% ethanol was based on its ability to optimize the extraction of both polar and apolar bioactive compounds, maximize extraction efficiency while minimizing toxicity, preserve biological activity of propolis, and align with widely used concentrations in the literature. Lower ethanol concentrations may limit the solubility of flavonoids and phenolic compounds, reducing extraction efficiency, while higher ethanol concentrations (above 50%) can lead to the degradation of heat- and solvent-sensitive bioactive components and increase cytotoxicity in biological applications [24,42]. In the literature, many studies have administered propolis extracts to mice at different doses and durations, with reported dosages including 160 mg/kg/day [31], 100 mg/kg/twice a week [43], 50 µg/kg/day [44], and 100 mg/kg/twice a week [45]. In our study, TG, TC, OxLDL, and LOX-1 levels in the serum of mice were higher in the Case group compared to all other groups, with a statistically significant difference (*p* < 0.004). Similar studies have reported significant decreases in TC and TG levels in all groups where EEP was administered [31,45,46]. Regarding OxLDL levels, previous research indicates that EEP reduces OxLDL-induced intracellular lipid accumulation and OxLDL uptake in RAW264.7 cells [47] and human umbilical vein endothelial cells (HUVEC) [48,49]. However, no study has specifically examined the effect of propolis on serum OxLDL levels in ApoE^−/−^ mice. For LOX-1 levels, it has been reported that niclosamide, an NF-κB inhibitor, decreases LOX-1 expression in carotid plaques and vascular smooth muscle cells (VSMCs) of ApoE^−/−^ mice, and that EEP inhibits LOX-1-mediated oxidative stress in HUVECs, thereby protecting them from OxLDL-induced damage [48,50,51]. No studies have investigated the effect of propolis on serum LOX-1 levels. Thus, our findings indicate that propolis extracts had a lowering effect on TG, TC, OxLDL, and LOX-1 levels. OxLDL levels in the aortic arches of mice were significantly higher in the Case group compared to all other groups (*p* < 0.004). In the literature, it has been reported that quercetin, a component of propolis, decreases OxLDL levels in ApoE^−/−^ mice, that TSG (2,3,5,4-tetrahydroxystilbene-2-O-β-D-glucoside) decreases OxLDL concentration in aortic tissue, and that CAPE, another propolis component, has antiproliferative effects on rat aortic VSMCs [52,53,54]. However, there are no studies investigating the effect of propolis and propolis-derived compounds on OxLDL levels in aortic arch tissue. The anti-atherosclerotic effects of propolis observed in this study may be attributed to its ability to reduce OxLDL and LOX-1 levels, thereby inhibiting endothelial dysfunction, oxidative stress, and inflammatory responses. Propolis-derived flavonoids and phenolic compounds likely exert their effects by modulating NF-κB and Mitogen-Activated Protein Kinases (MAPK) signaling pathways, reducing macrophage foam cell formation, and enhancing antioxidant defense mechanisms, ultimately contributing to the stabilization of atherosclerotic plaques [24,42]. The possible effects of WEP and EEP on plaque burden in the aortic root were examined histologically by semi-quantitative ORO staining. Atherosclerotic plaque burden was significantly higher in the Case group compared to the WEP and EEP groups (*p* < 0.05). In the literature, both WEP and EEP have been reported to reduce atherosclerotic plaque burden. Additionally, lipid accumulation in the tunica intima of the aortic root was lower in the WEP and EEP groups compared to the Case group, with a statistically significant difference observed (*p* < 0.05). The fact that these findings correlate with serum TC and TG levels further supports our study [30,31]. Although the ethanol group showed a significant reduction in vascular adipocyte accumulation compared to the case group, this effect is likely not due to ethanol itself but rather limited to potential mild anti-inflammatory effects, whereas the greater reduction observed in the EEP group supports the role of bioactive compounds of propolis in regulating lipid accumulation and inflammation. While both extracts demonstrated anti-atherosclerotic effects, EEP may be preferred for its stronger pharmacological activity and higher bioavailability, whereas WEP offers a safer, alcohol-free option suitable for broader use, and future studies are needed to determine the most appropriate extract for complementary supplementation. The use of propolis samples from different regions rather than a single source enhances the chemical diversity and biological efficacy of the extracts, increases the generalizability and reliability of the findings, minimizes natural variations, and provides a more realistic model for evaluating its potential as a pharmaceutical or dietary supplement in atherosclerosis treatment [42,55].

## 5. Conclusions

This study is the first to examine the effects of water and ethanolic extracts of propolis on OxLDL and LOX-1 levels in ApoE^−/−^ mice fed a HFD. Propolis was found to reduce dyslipidemia, oxidative stress, and inflammatory parameters, demonstrating its anti-inflammatory, antioxidant, and anti-atherogenic properties. These findings suggest that propolis extracts may serve as a complementary supplement in the treatment of atherosclerosis, obesity, diabetes, and various CVD. However, since this study was conducted using an animal model, its applicability to humans should be validated through clinical trials. Further long-term research is needed to assess its sustained effects, elucidate its molecular and cellular mechanisms, and determine the efficacy of oral administration and different dosage regimens.

## Figures and Tables

**Table 1:** Details of Experimental Groups and Intervention Procedures.

Group (*n* = 8)	Animal Type	Diet	Time	Injection
Control	C57BL-6J	Control Diet (CD)	16 Weeks	
Case	ApoE^−/−^	HFD	16 Weeks	
Sham	C57BL-6J	HFD	16 Weeks	
WEP	ApoE^−/−^	HFD	16 Weeks	16 Weeks 400 mg/kg WEP by intraperitoneal (i.p.) injection every day for the last 4 weeks
EEP	ApoE^−/−^	HFD	16 Weeks	16 Weeks 200 mg/kg EEP by i.p. injection every day for the last 4 weeks
Ethanol	ApoE^−/−^	HFD	16 Weeks	Ethanol 30% by i.p. injection every day for the last 4 weeks

**Table 2:** Serum TG and TC levels.

Parameters	Control (C57BL/6J + CD)	Case (ApoE^−/−^ + HFD)	Sham (ApoE^−/−^ + HFD)	WEP (ApoE^−/−^ + HFD)	EEP (ApoE^−/−^ + HFD)	Ethanol (ApoE^−/−^ + HFD)
TG (mg/dL) (Median-IQR)	82 (72–82)	144 ^a^ (110–117)	99 ^b^ (70–79)	64 ^a,b^ (52–58)	67 ^a,b^ (54.5–62)	137 ^a^ (92.3–1013)
TC (mg/dL) (Median-IQR)	115 (128–154)	1569 ^a^ (1355–1412)	164 (129–138)	1106 ^a,b^ (1212–1267)	1148 ^a,b^ (1103–1259)	1610 ^a^ (1327–1505)

Results expressed as median-IQR. ^a^: Statistically significant difference compared to Control group (*p* < 0.004), ^b^: Statistically significant difference compared to the Case group (*p* < 0.004).

**Table 3:** Aortic root histopathological damage scoring findings (AHHS, Median- IQR).

Group	Adipocyte Subendothelial Involvement	Adipocyte Vascular Wall Involvement	Fibrosis Cap Formation	AHHS
Control	0 (0–0)	0 (0–0)	0 (0–0)	0 (0–0)
Case	2 (2–2) ^a^	1.5 (1–1) ^a^	0 (0–0.5)	3 (2–3) ^a^
Sham	2 (2–3) ^b^	1 (0–1) ^b^	1 (0–1) ^b^	3 (2–3) ^b^
WEP	1 (0.5–1) ^a,b^	0.5 (0–1) ^a,b^	0 (0–0) ^a,b^	1 (0.5–1) ^a,b^
EEP	1 (0–1) ^a,b^	0 (0–1) ^a,b^	0 (0–0) ^a,b^	0.5 (0–1) ^a,b^
Ethanol	0 (0–0) ^a,b^	1 (0–0) ^a,b^	0 (0–0) ^a,b^	0 (0–1) ^a,b^

When evaluated using the Kruskal–Wallis/Tamhane tests; a: Indicates a significant difference compared to the Control group (*p* < 0.05), b: Indicates a significant difference compared to the Case group (*p* < 0.05).

## Data Availability

All data generated or analyzed during this study are included in this published article.
